# Repetitive Transcranial Magnetic Stimulation of the mPFC Ameliorates Stress‐Induced Hyperalgesia by Alleviating Microglia Activation in Rats

**DOI:** 10.1002/brb3.71635

**Published:** 2026-07-28

**Authors:** Jian Qi, Chen Chen, Qian Gao, Lu Li

**Affiliations:** ^1^ Department of Orthopedics The 960th Hospital of PLA Jinan China; ^2^ Department of Pharmacy The Second Hospital of Shandong University Jinan China; ^3^ Jinzhou Medical University, The 960th Hospital of the Chinese People's Liberation Army Joint Logistic Support Force Postgraduate Training Base Jinan China

**Keywords:** complete Freund's adjuvant, medial prefrontal cortex, microglia, rat, repetitive transcranial magnetic stimulation, single‐prolonged stress, stress‐induced hyperalgesia

## Abstract

**Purpose:**

Repetitive transcranial magnetic stimulation (rTMS) is a noninvasive neuromodulation technique that uses magnetic fields to stimulate or inhibit specific populations of neurons in the brain. rTMS targeting the cortex has emerged as an effective treatment for chronic pain. It is established that stress can heighten pain sensitivity, a condition termed stress‐induced hyperalgesia (SIH). However, neither direct evidence for rTMS treatment of SIH nor a clear understanding of its therapeutic mechanisms has been established.

**Methods:**

In this article, we studied the effectiveness of rTMS in SIH using a rat CFA (complete Freund's adjuvant) + SPS (single‐prolonged stress)‐induced model. Following SPS + CFA treatment, a significant enhancement in both medial prefrontal cortex (mPFC) microglia activation by immunohistochemistry and Western blot analyses and mechanical allodynia was observed, as compared with the application of SPS or CFA alone. Compared to the untreated SPS + CFA model group, 10 Hz rTMS treatment significantly elevated the paw withdrawal threshold. This effect was also significantly greater than that observed in the group receiving 1 Hz rTMS. Moreover, the application of 10 Hz rTMS inhibited microglial activation in the SPS + CFA model, along with alleviating associated anxiety‐like behaviors.

**Finds and Conclusion:**

These results suggest that rTMS might have an effect on the treatment of SIH.

## 1 Introduction

Pain is not a simple sensation but a multifaceted experience, woven together from sensory, emotional, and cognitive threads that shape how we perceive, react to, and interpret injury or threat (Liu and Chen 2014). Chronic pain rarely occurs in isolation; it frequently coexists with emotional and cognitive disturbances that not only erode patients’ well‐being but may also intensify the sensory experience of pain. Moreover, chronic pain and stress share a reciprocal link: Exposure to psychological or physical stressors has been shown to heighten pain sensitivity—a process known as stress‐induced hyperalgesia (SIH) (Imbe et al. 2006). Post‐traumatic stress disorder (PTSD) is a psychologically crippling illness triggered by trauma, characterized by a cluster of debilitating symptoms—with severe anxiety standing out as a core component (Harpviken et al. 2025). Emerging evidence suggests that PTSD may heighten pain sensitivity, further entangling the psychological and physiological toll it takes on those affected (Sepehrinezhad and Gorji 2026).

Within the brain, the mesocorticolimbic circuitry has been shown to represent both the negative affective dimension of pain and the positive hedonic experience of pain cessation (Navratilova and Porreca 2014). Neuroimaging reveals that pain doesn't just trigger sensory pathways; it also recruits core reward‐related regions such as the PFC, nucleus accumbens, and ventral tegmental area (Wanigasekera et al. 2012). The PFC exercises top‐down executive authority over a range of vital operations, including learning, memory, and the blending of sensory with emotional experience (Arnsten et al. 2012). The medial prefrontal cortex (mPFC) plays a predominant role in coding the affective facet of nociceptive experience, particularly the subjective sense of unpleasantness associated with pain (Lorenz et al. 2002). Cognitive behavioral therapy has been linked to elevated mPFC activation among chronic pain sufferers (Jensen et al. 2012).

The single‐prolonged stress (SPS) paradigm stands as a well‐validated experimental approach in PTSD research, capable of generating key phenotypic features of the disease for mechanistic study (Qi et al. 2014; Mifsud et al. 2011; Wang et al. 2008). Although anxiety in PTSD is known to exacerbate pain sensitivity, achieving satisfactory therapeutic outcomes for this dual burden presents a significant clinical challenge, highlighting the need for novel treatment strategies. Recent evidence suggests that pain relief can be achieved through transcranial magnetic stimulation (TMS) or deep brain stimulation (DBS) targeting the anterior cingulate cortex (ACC) in certain patient populations (Mohseni et al. 2012). Moreover, accumulating evidence indicates that high‐frequency (10 Hz) repetitive transcranial magnetic stimulation (rTMS) targeting the right dorsolateral prefrontal cortex confers symptomatic benefit in PTSD, representing a well‐tolerated and noninvasive treatment modality (Wang et al. 2015; Fox et al. 2014).

Serving as the central nervous systems (CNS's) frontline immune cells, microglia are key arbiters of synaptic plasticity throughout pain signaling (Feng et al. 2024). Microglia residing in the mPFC not only support maternal behavior but also serve a protective function against the lasting effects of stress experienced during adolescence (Chen et al. 2025), and microglial activation serves as a key mediator of stress‐induced synaptic elimination within cortical layer two/three of the mPFC in male mice (Tillmon et al. 2024). It remains unknown whether rTMS can modulate SIH via the mPFC, what stimulation parameters are effective, and how microglia are involved in this regulatory process.

To investigate the effects of rTMS on SIH, we employed two validated rodent models. The persistent inflammatory pain model was induced via unilateral right hindpaw injection of complete Freund's adjuvant (CFA; an antigen emulsion in mineral oil). For PTSD‐like symptoms, the SPS protocol was implemented, which comprised the following sequential stressors: 2‐h physical restraint, immediate 20‐min forced swimming, 15‐min recovery, and exposure to diethyl ether until loss of consciousness. On the basis of these prior observations, we, therefore, developed a combined SPS + CFA paradigm to directly examine the interaction between PTSD‐like stress and chronic inflammatory pain. This integrated model enables the evaluation of rTMS effects on stress‐related hyperalgesia and anxiety behavior (Qi et al. 2014, 2016). In this study, we hypothesized that rTMS could serve as a potential treatment for SIH. Furthermore, we aimed to examine whether microglial activity mediates the effects of rTMS on the interaction between nociceptive and psychological stress pathways, which may underlie their shared mechanisms in promoting hyperalgesia.

## 2 Materials and Methods

### 2.1 Animals

All experimental protocols were approved by the Animal Ethics Committee of the 960th Hospital of the People's Liberation Army (Jinan, China). Adult male Sprague‐Dawley rats weighing 250–300 g were used in this study. In accordance with the *Guidelines for the Care and Use of Mammals in Neuroscience and Behavioral Research*, stringent measures were taken to minimize animal suffering and to reduce the number of animals used. Throughout the study, rats were housed in pairs under a reversed 12‐h light/dark cycle (lights off at 8:00 a.m.) and provided with ad libitum access to food and water.

### 2.2 Experimental Design and Protocol

**FIGURE 1:**
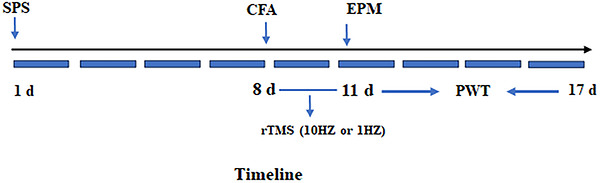
Overlap of behavioral tests done (EPM and PWT), the time course of SPS exposure, time course for CFA injection, and the time for rTMS treatment. CFA, complete Freund's adjuvant; EPM, elevated plus maze; PWT, paw withdrawal threshold; rTMS, repetitive transcranial magnetic stimulation; SPS, single‐prolonged stress.

A total of 102 animals were included in the study and randomly allocated into the following groups: control, CFA, SPS, SPS + CFA, SPS + CFA with repetitive 1 Hz TMS, and SPS + CFA with repetitive 10 Hz TMS (see Figure 1 for experimental design). Magnetic stimulation was delivered using a high‐frequency magnetic stimulator equipped with a 4 cm diameter circular coil placed above the rat's head. The auditory aspect of rTMS was simulated with a computer‐controlled subwoofer system that matched the frequency range and sound level of genuine rTMS. On Day 1, rats were subjected to SPS exposure. On Day 8, CFA was injected following the same protocol as our previous study (Qi et al. 2026); notably, rats in the SPS + CFA group underwent SPS exposure before receiving the CFA injection. From Days 8 to 11, animals received daily rTMS treatments (10 or 1 Hz, 3 s per on/off cycle for 20 min) (total stimulation time) targeting the frontal cortex for 10 consecutive days. Paw withdrawal threshold (PWT) was evaluated in a double‐blind fashion daily at a fixed time between Days 11 and 17, whereas the elevated plus maze (EPM) test was conducted under double‐blind conditions on Day 11. All behavioral testing was conducted under double‐blind conditions, with sessions commencing at a fixed time each day. Before each behavioral assessment, rats were allowed a 15‐min acclimation period in the testing environment. For the rats designated for immunohistochemical staining and Western blot analysis, the experiment was terminated on Day 11, and they were euthanized without undergoing the EPM procedure.

### 2.3 Behavioral Tests

#### 2.3.1 Mechanical Sensitivity Testing

For the assessment of evoked reflex responses, animals were positioned on a mesh grid inside a transparent plastic enclosure. Mechanical sensitivity was tested by applying von Frey filaments (Stoelting Co., USA) to the mid‐plantar region of the ipsilateral paw. Each filament was pressed upward until it bent, and five applications were performed per filament in ascending order of force. The PWT was determined as the minimum force (in grams) required to elicit a withdrawal response in ≥50% of the trials. The presence of any of the following behaviors during acute withdrawal was recorded as a positive sign: biting, licking, or shaking of the ipsilateral hind limb, as well as vocalization. Detailed methodological procedures can be found in our previous studies (Qi et al. 2014, 2016).

#### 2.3.2 Elevated Plus Maze

The EPM consisted of two open arms (30 × 5 × 0.5 cm^3^) and two enclosed arms (30 × 5 × 15 cm^3^), which were positioned opposite each other and connected by a central platform (5 × 5 cm^2^). Each rat was gently placed on the central platform and allowed to freely explore the apparatus for 5 min. The behavior was recorded and subsequently analyzed using a video tracking system (Shanghai Mobile Datum Technology Co. Ltd., China). The recorded data were used to calculate the total time spent in the open arms, the total distance traveled in the open arms, and the number of entries into the open arms. Detailed methodological procedures can be found in our previous studies (Qi et al. 2014, 2016).

#### 2.3.3 Immunohistochemistry

Rats were euthanized and transcardially perfused with phosphate‐buffered saline (PBS; pH 7.4) followed by 10% formalin fixative solution. Coronal brain sections of 50 µm thickness were prepared and stained with primary anti‐GFAP antibody (Rabbit anti‐GFAP, 1:500, Abcam, ab68428, UK) and secondary antibody (Donkey anti‐rabbit Alexa 488, 1:400, Molecular Probes). The antibody incubation medium consisted of 5% (v/v) NFBS, 0.3% (v/v) Triton X‐100, 0.05% (w/v) NaN_3_, and 0.25% (w/v) carrageenan in 0.01 M PBS (PBS‐NFBS, pH 7.4). All sections were washed three times in PBS and subsequently imaged using a confocal laser scanning microscope (FV1000 Olympus, Tokyo, Japan). Detailed methodological procedures can be found in our previous studies (Qi et al. 2016, 2026).

#### 2.3.4 Western Blot Analysis

Rats were deeply anesthetized via intraperitoneal injection of sodium pentobarbital (60 mg/kg) dissolved in 0.9% (w/v) saline. Following loss of consciousness, rats were rapidly sacrificed by decapitation, and the mPFC was quickly dissected on an ice‐cold plate. Tissue samples were homogenized in ice‐cold RIPA lysis buffer (50 mM Tris‐HCl, pH 7.4, 150 mM NaCl, 1% NP‐40, 0.5% sodium deoxycholate, and 0.1% SDS) supplemented with protease and phosphatase inhibitor cocktails (Thermo Fisher Scientific, Waltham, MA, USA). Homogenates were sonicated briefly and then centrifuged at 12,000 × *g* for 20 min at 4°C. The supernatant was collected, and protein concentrations were determined using a bicinchoninic acid (BCA) protein assay kit (Pierce, Rockford, IL, USA). Equal amounts of protein (50 µg per lane) from each sample were mixed with Laemmli sample buffer containing 5% β‐mercaptoethanol, heated at 95°C for 5 min, and resolved by 10% sodium dodecyl sulfate–polyacrylamide gel electrophoresis (SDS–PAGE) at 120 V for approximately 90 min. Separated proteins were then electrophoretically transferred onto polyvinylidene fluoride (PVDF) membranes (Immobilon‐P, Millipore, Billerica, MA, USA) using a wet transfer system (Bio‐Rad, Hercules, CA, USA) at 100 V for 1 h at 4°C. Prior to use, PVDF membranes were activated in methanol for 30 s and rinsed in transfer buffer. After transfer, membranes were blocked in Tris‐buffered saline containing 0.1% Tween‐20 (TBST) and 5% nonfat dry milk (Bio‐Rad) for 1 h at room temperature on a shaker. Following blocking, membranes were incubated overnight at 4°C with primary antibodies: rabbit anti‐GFAP (1:1000; Abcam, Cambridge, MA, USA) and mouse anti‐β‐actin (1:1000; Sigma‐Aldrich, St. Louis, MO, USA) as a loading control. All primary antibodies were diluted in TBST containing 5% bovine serum albumin (BSA). The next day, membranes were washed three times with TBST (10 min each) and then incubated with horseradish peroxidase (HRP)‐conjugated secondary antibodies: anti‐rabbit IgG (1:3000) and anti‐mouse IgG (1:5000; both from Amersham Pharmacia Biotech Inc., Piscataway, NJ, USA) diluted in 5% non‐fat dry milk in TBST for 1 h at room temperature. After three additional washes with TBST (10 min each), immunoreactive bands were visualized using enhanced chemiluminescence (ECL) detection reagent (Amersham, Little Chalfont, UK) following the manufacturer's instructions. Chemiluminescent signals were captured using a digital imaging system (Bio‐Rad ChemiDoc XRS). Band intensities were quantified using Labworks Software (Ultra‐Violet Products, Cambridge, UK). The expression level of GFAP in each sample was normalized to the corresponding β‐actin signal, and all data were expressed as relative optical density values.

#### 2.3.5 Statistical Analysis

Mechanical allodynia data were analyzed using two‐way analysis of variance (ANOVA) (group × day). Behavioral data (from the EPM test), immunofluorescence intensity of GFAP, and GFAP protein expression levels were compared across groups using one‐way ANOVA. The Student–Newman–Keuls post hoc test was subsequently applied to assess intergroup differences. All data are presented as mean ± SEM. All statistical tests were two‐sided, and a *p* value less than 0.05 was considered statistically significant.

## 3 Results

### 3.1 SPS + CFA Treatment Induced Microglial Activation in the mPFC

**FIGURE 2:**
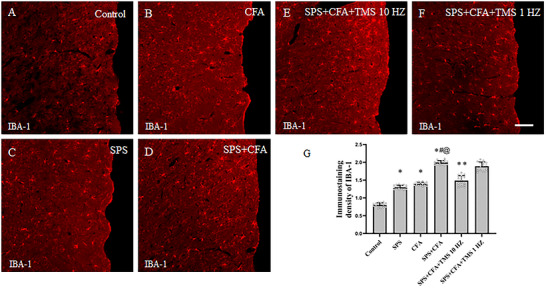
Immunofluorescent labeling of IBA‐1 in the mPFC by CFA, SPS, SPS + CFA, and rTMS treatment (A–F). Part (G) showing the statistical results of immunostaining density of IBA‐1 (*p < 0.05 vs. the control group; #*p* < 0.05 vs. the CFA group; @*p* < 0.05 vs. the SPS group; **p < 0.05 < SPS + CFA group). Scale bars = 100 µm. Results are expressed as the mean ± SEM. CFA, complete Freund's adjuvant; SPS, single‐prolonged stress; TMS, transcranial magnetic stimulation.

**FIGURE 3:**
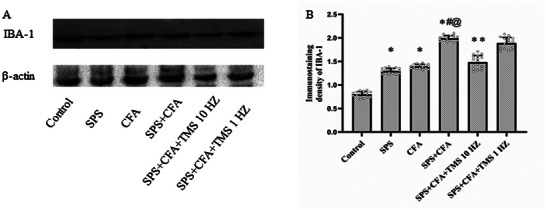
(A) Immunoblots of IBA‐1 in the mPFC by CFA, SPS, SPS + CFA, and rTMS treatments. (B) Densitometry analysis of western blot bands of IBA‐1. Compared to the control group, the protein of IBA‐1 was up in CFA, SPS and SPS + CFA exposure rats (*p < 0.05 vs. the control group; #*p* < 0.05 vs. the CFA group; @*p* < 0.05 vs. the SPS group; **p < 0.05 vs. the SPS + CFA group). The data are presented as mean ± SEM. CFA, complete Freund's adjuvant; SPS, single‐prolonged stress; TMS, transcranial magnetic stimulation.

To assess the impact of CFA combined with SPS on microglial activation in the mPFC, we examined Iba‐1 immunoreactivity in the ipsilateral cortex following treatments with vehicle, CFA alone, SPS alone, and CFA + SPS. A one‐way ANOVA showed statistically significant differences in Iba‐1 immunopositive density in the ipsilateral mPFC among the groups (*F*_3, 12_ = 76.55, *p* < 0.001). Quantitative analysis demonstrated that Iba‐1‐positive immunoreactive density was significantly increased in the CFA, SPS, and CFA + SPS groups compared to the control group (*p* < 0.05). Notably, the CFA + SPS group exhibited a significantly greater density than either the CFA or SPS group alone (*p* < 0.05) (Figure 2). The levels of Iba‐1 in the mPFC were also analyzed. Significant differences in Iba‐1 protein levels in the mPFC were observed among the groups (*F*_3, 12_ = 157.6, *p* < 0.001). Western blot analysis revealed an upregulation of Iba‐1 in SPS‐, CFA‐, and SPS + CFA‐exposed rats compared to naïve rats (*p* < 0.05). Furthermore, Iba‐1 levels were significantly higher in SPS + CFA‐exposed rats than in those exposed to either CFA alone (*p* < 0.05) or SPS alone (*p* < 0.05) (Figure 3).

### 3.2 Effects of Different rTMS Frequencies on Microglial Expression in the mPFC Following SPS + CFA

To investigate whether rTMS modulates microglial expression and to identify an effective stimulation frequency, we assessed Iba‐1 levels in the mPFC using morphological analysis and Western blotting. Our data revealed that the density of Iba‐1 positive immunostaining in the ipsilateral mPFC was significantly decreased following treatment with 10 Hz rTMS compared to the CFA + SPS group (*p* < 0.05). In contrast, 1 Hz rTMS showed no significant effect on Iba‐1 immunopositive density in the ipsilateral mPFC after SPS + CFA exposure (Figure 2). To further examine the protein levels of microglial activation, we performed Western blot analysis on mPFC tissue samples from rats in the CFA + SPS, rTMS (1 Hz), and rTMS (10 Hz) groups. The results showed that Iba‐1 expression was significantly elevated in SPS + CFA rats. Following treatment with 10 Hz rTMS, Iba‐1 protein levels were significantly reduced compared to those in the SPS + CFA group (*p* < 0.05). In contrast, treatment with 1 Hz rTMS did not result in a significant decrease in Iba‐1 protein levels compared to the SPS + CFA group (*p* > 0.05) (Figure 3).

### 3.3 Effects of Different rTMS Frequencies on Anxiety‐Like Behavior Induced by SPS + CFA in the EPM Test

**FIGURE 4:**
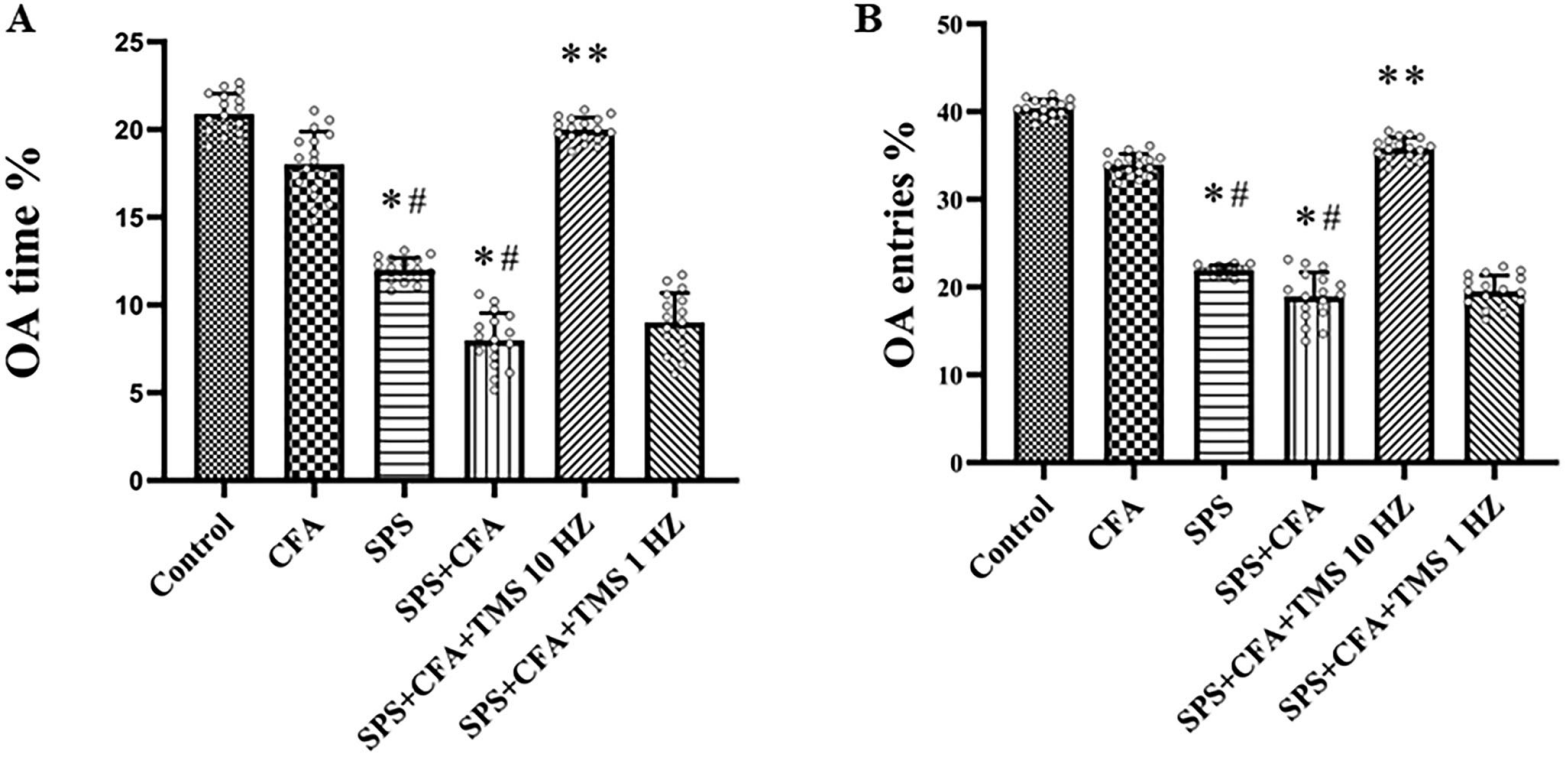
(A) The percentage of open‐arm time (open‐arm (OA) time/total time) was shown in this histogram in different groups and rTMS treatment in the EPM test (*p < 0.05 vs. the control group;**p < 0.05 vs. the CFA group; #*p* < 0.05, compared to SPS + CFA group), and (B) the percentage of open‐arm entries (open‐arm (OA)entries/total entries) was shown in this histogram in different groups in the EPM test (*p < 0.05 vs. the control group;**p < 0.05 vs. the CFA group; #*p* < 0.05, compared to SPS + CFA group). Results are expressed as the mean ± SEM. CFA, complete Freund's adjuvant; SPS, single‐prolonged stress; TMS, transcranial magnetic stimulation.

We used the EPM to assess anxiety‐like behavior induced by SPS, CFA, and SPS + CFA, quantifying the percentage of time spent in open arms (OA time%) and the percentage of open‐arm entries (OA entries%). Statistical analysis via one‐way ANOVA demonstrated significant group differences in both OA time% (*F*_(11, 79)_ = 57.18, *p* < 0.001) and OA entries% (*F*_(11, 79)_ = 86.17, *p* < 0.001). Rats exposed to SPS + CFA or SPS alone showed significantly reduced OA time% and OA entries% compared to control or CFA rats (Student–Newman–Keuls test, *p* < 0.05). In subsequent rTMS intervention, 10 Hz stimulation further decreased these anxiety‐like behaviors versus the SPS + CFA group (*p* < 0.05), whereas 1 Hz stimulation showed no significant effect (*p* > 0.05) (Figure 4).

### 3.4 Effects of Different rTMS Frequencies on SPS + CFA‐Induced Hyperalgesia

**FIGURE 5:**
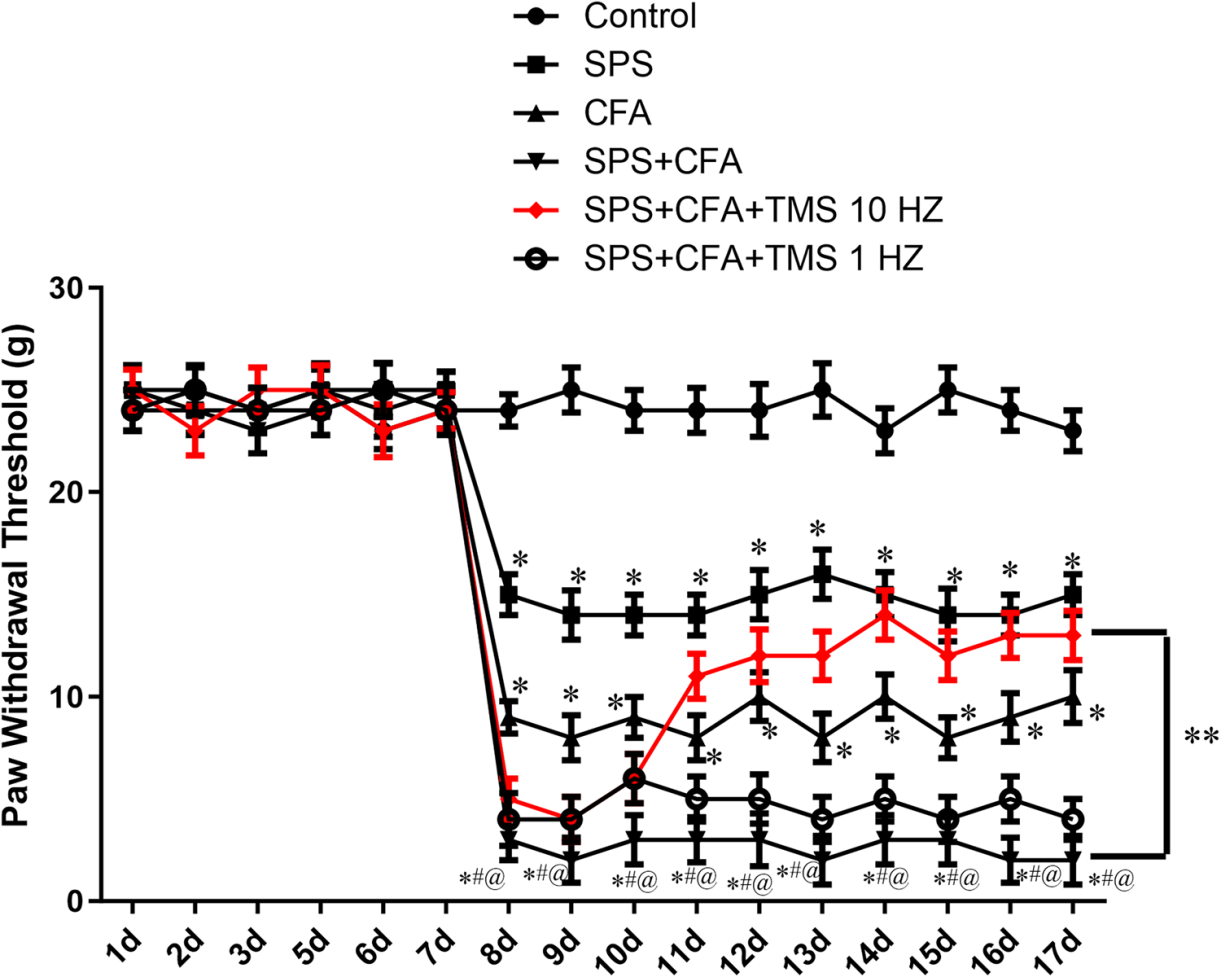
Effect of CFA, SPS, CFA +  SPS and TMS treatment on mechanical hyperalgesia. Von Frey tests showed that SPS + CFA exposure rats had significantly lower mechanical hyperalgesia. Compared with the control rats, the PWTs were decreased in the injured hindpaw of the CFA, SPS, and SPS + CFA exposure rats. SPS + CFA group had significantly reduced PWTs relative to both the SPS group (*p* < 0.05) and the CFA group (*p* < 0.05). rTMS 10 HZ treatment reversed the PWT reduction (*p < 0.05, compared to the control group; #*p* < 0.05, compared to the SPS group; @*p* < 0.05, compared to the CFA group; and **p < 0.05 compared to SPS + CFA group. Comparison with Students–Newman–Keuls test). CFA, complete Freund's adjuvant; SPS, single‐prolonged stress; TMS, transcranial magnetic stimulation.

The present analysis, consistent with previous findings, showed a significant overall effect on PWTs (*F*_(6, 324)_ = 88.57, *p* < 0.001) (Qi et al. 2014, 2016; Aad et al. 2010; Zhang et al. 2020). Compared to control rats, PWTs were significantly lower from Day 8 in the SPS (*p* < 0.05), CFA (*p* < 0.05), and SPS + CFA (*p* < 0.01) groups, with the latter showing the most pronounced reduction in hindpaw pain threshold. It was demonstrated that the SPS + CFA group had significantly reduced PWTs relative to both the SPS group (*p* < 0.05) and the CFA group (*p* < 0.05). rTMS treatment of 10 Hz significantly increased the PWT in both SPS + CFA‐exposed and SPS‐exposed rats (*p* < 0.05). In contrast, 1 Hz rTMS produced no significant increase in PWT in either of these groups (*p* > 0.05) (Figure 5).

## 4 Discussion

Stress also has the capacity to heighten pain sensitivity—a phenomenon termed SIH. In fact, a wide array of stressors, such as repeated forced swimming, acute or prolonged restraint, unfamiliar surroundings, horizontal rotation, and social defeat, have all been shown to elicit hyperalgesia in animal studies (Jennings et al. 2014). This process goes far beyond mere hyperreflexia; for instance, psychological factors, such as anxiety or the mere expectation of pain, have been demonstrated to amplify pain sensitivity in humans, whereas stress is broadly acknowledged to exacerbate chronic pain in clinical settings.

Recent years have seen neurostimulation widely adopted in treating various psychiatric illnesses. TMS, which modulates brain activity without invasive procedures, now serves a key function in easing the symptom burden of PTSD and anxiety (Lantrip et al. 2022). Studies on TMS for PTSD have produced mixed results, largely due to small sample sizes. Although its positive effects on mood, anxiety, and stress‐related disorders are well documented, whether TMS can relieve SIH remains unknown. It should also be noted that TMS includes multiple technical variants; the most common, rTMS, works by repeatedly applying magnetic pulses to achieve clinical benefits (Rossi et al. 2009). The working principle of TMS involves electromagnetic induction to influence neural firing. This mechanism enables direct stimulation of neural tissue, which, in turn, provides indirect control over cortical activity. Furthermore, when different frequencies are applied, the resulting effects vary according to the brain region under stimulation (Edinoff et al. 2022). At present, only two rTMS protocols have received clinical approval: One involves high‐frequency (≥5 Hz) stimulation applied to the left DLPFC to treat major depressive disorder (MDD), and the other uses low‐frequency (<5 Hz) stimulation directed at the supplementary motor area for managing obsessive‐compulsive disorder (OCD) (Zorzo et al. 2019; Iglesias 2020). Although previous studies have suggested that rTMS therapy may reduce pain in patients with neuropathic pain—as measured by tools such as the VAS or NRS—the overall evidence remains inconclusive, largely because findings have been inconsistent across different investigations (Anderson et al. 2020). We found that in animal models of CFA‐induced inflammation and anxiety‐like behavior, daily sessions of 10 Hz rTMS effectively counteracted SIH and the accompanying expression of anxiety‐like behaviors in male rats. The existing literature clearly demonstrates that male and female rodents differ fundamentally in their analgesic responses—whether from administered opiates, endogenous opioids, or stress. Typically, males show more pronounced analgesia, a pattern shaped primarily by the organizational effects of gonadal hormones, with activational effects playing a secondary role (Pizaña‐Encarnación et al. 2025). A limitation of this study is that we used only male rats to normalize experimental conditions. Future work should address sex differences by including females.

TMS stimulates the cerebral cortex noninvasively by sending magnetic pulses across the skull. Reaching about 2 cm deep, these pulses induce localized electrical currents in cortical neurons. Clinicians can tailor TMS therapy by adjusting two key parameters: The stimulation frequency and the specific cortical site being targeted (Edinoff et al. 2022; Iglesias 2020). The mPFC plays a key role in emotional regulation, and its activity becomes dysregulated in a range of conditions, including anxiety disorders, depression, and PTSD. As a result, interventions that can modulate mPFC function offer promise as transdiagnostic treatments for psychiatric illness (Klune et al. 2021). This study was designed to test whether rTMS produces its therapeutic effects in SIH through causal modulation of mPFC activity. Our findings revealed that Iba‐1‐positive immunoreactive density was significantly elevated in the CFA, SPS, and CFA + SPS groups relative to controls, with the CFA + SPS group showing the highest density among all experimental conditions. Application of 10 Hz rTMS significantly reduced Iba‐1 protein levels compared to the untreated CFA + SPS group, implicating mPFC microglial activation in the mechanism underlying high‐frequency rTMS. That said, prior work has identified the DLPFC as a promising target for neuromodulation aimed at alleviating mood symptoms in PTSD patients (Berlim and Van Den Eynde 2014). The discrepancy between our findings and those of earlier studies may stem from the limited penetration depth of TMS. Although TMS typically reaches only about 2 cm, producing focal stimulation of cortical neurons, it could still indirectly influence deeper brain regions—such as the mPFC—via functional network connections. That said, the precise mechanisms underlying this effect remain unclear, and further research is needed to fully understand this phenomenon. In addition, microglia, the resident immune cells of the CNS, act as both sensors of neuronal activity and regulators of essential physiological brain functions. Previous studies have shown that 10 Hz rTMS can induce synaptic plasticity in organotypic slice cultures and anesthetized mice—a process that depends on microglia and plasticity‐promoting cytokines, despite the absence of observable changes in microglial shape (Eichler et al. 2023). The present study employed morphological and Western blot analyses to demonstrate that 10 Hz rTMS significantly inhibits the SPS + CFA‐induced elevation of both Iba‐1‐positive immunoreactive density and Iba‐1 protein levels in rats. These preliminary observations extend the scope of previous research. Our morphological and Western blot data from rats show that 10 Hz rTMS suppresses SPS + CFA‐induced increases in Iba‐1‐positive density and Iba‐1 protein levels, offering preliminary evidence that extends prior findings. Yet the underlying mechanisms remain unclear: Does this effect depend on microglia‐derived cytokines that shape synaptic plasticity, or does it involve other, as‐yet‐unknown forms of microglial adaptation? Further research is needed to resolve this question.

In summary, our findings indicate that rTMS alleviates SIH in rats by suppressing microglial activation within the mPFC. This preliminary evidence supports the notion that rTMS treatment parameters could be refined and optimized on the basis of dynamic changes in microglial adaptation.

## Author Contributions

**Qian Gao**: methodology. **Jian Qi**: conceptualization, methodology, software, data curation, supervision, formal analysis, validation, funding acquisition, investigation, writing – original draft, visualization, writing – review and editing, project administration, resources. **Chen Chen**: software, formal analysis, writing – original draft. **Lu Li**: methodology, investigation.

## Conflicts of Interest

The authors declare no conflicts of interest.

## Data Availability

The data that support the findings of this study are available from the first author, Jian Qi, upon reasonable request.
